# *Cis*-mediated interactions of the SARS-CoV-2 frameshift RNA alter its conformations and affect function

**DOI:** 10.1093/nar/gkac1184

**Published:** 2022-12-20

**Authors:** Lukas Pekarek, Matthias M Zimmer, Anne-Sophie Gribling-Burrer, Stefan Buck, Redmond Smyth, Neva Caliskan

**Affiliations:** Helmholtz Institute for RNA-based Infection Research (HIRI-HZI), Würzburg, Germany; Helmholtz Institute for RNA-based Infection Research (HIRI-HZI), Würzburg, Germany; Helmholtz Institute for RNA-based Infection Research (HIRI-HZI), Würzburg, Germany; Helmholtz Institute for RNA-based Infection Research (HIRI-HZI), Würzburg, Germany; Helmholtz Institute for RNA-based Infection Research (HIRI-HZI), Würzburg, Germany; Medical Faculty, Julius-Maximilians University Würzburg, Würzburg, Germany; Helmholtz Institute for RNA-based Infection Research (HIRI-HZI), Würzburg, Germany; Medical Faculty, Julius-Maximilians University Würzburg, Würzburg, Germany

## Abstract

The RNA genome of SARS-CoV-2 contains a frameshift stimulatory element (FSE) that allows access to an alternative reading frame through −1 programmed ribosomal frameshifting (PRF). −1PRF in the *1a/1b* gene is essential for efficient viral replication and transcription of the viral genome. −1PRF efficiency relies on the presence of conserved RNA elements within the FSE. One of these elements is a three-stemmed pseudoknot, although alternative folds of the frameshift site might have functional roles as well. Here, by complementing ensemble and single-molecule structural analysis of SARS-CoV-2 frameshift RNA variants with functional data, we reveal a conformational interplay of the 5′ and 3′ immediate regions with the FSE and show that the extended FSE exists in multiple conformations. Furthermore, limiting the base pairing of the FSE with neighboring nucleotides can favor or impair the formation of the alternative folds, including the pseudoknot. Our results demonstrate that co-existing RNA structures can function together to fine-tune SARS-CoV-2 gene expression, which will aid efforts to design specific inhibitors of viral frameshifting.

## INTRODUCTION

**Figure 1.**
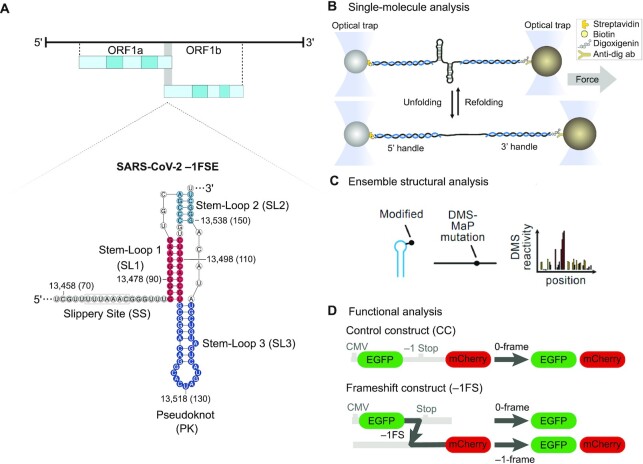
An integrated system for probing the structural landscape of the SARS-CoV-2 FSE. (**A**) Schematic representation of the SARS-CoV-2 programmed −1 ribosomal frameshifting element within the SARS-CoV-2 genome. The secondary structure of the FSE is derived from published structural models (12,13). SL1 (in magenta), SL2 (in light blue) and SL3 (in dark blue) with the corresponding genomic nucleotide positions indicated; numbers in brackets refer to the position relative to the longest RNA variant measured in this study (FSE-V4). (**B**) Schematic illustration of optical tweezers experiments. RNA is hybridized to single-stranded DNA handles flanking the SARS-CoV-2 frameshift site and conjugated to functionalized beads. A focused laser beam is used to exert pulling force from one end of the molecule. The force is gradually increased until the RNA fully unfolds (bottom). (**C**) Structural profiles are obtained by DMS-MaP, in which DMS preferably reacts with unpaired A and C residues, which are later read out as mutations and converted into DMS reactivities by comparing DMS-treated samples vs. untreated controls. (**D**) Scheme of the dual-fluorescence frameshift reporter construct. EGFP and mCherry are separated by a StopGo signal as well as by a stop codon in-frame with EGFP. As a result, translation in 0-frame produces only EGFP, whereas translation in −1-frame produces both EGFP and mCherry. The ratio of mCherry to EGFP fluorescence normalized to a control lacking the frameshift signal encoding both EGFP and mCherry is used to quantify frameshift efficiencies (FE). See also Materials and Methods.

Many viruses, including Coronaviruses, employ several recoding strategies that allow access to an overlapping and functional reading frame, thereby increasing the regulatory potential and the coding repertoire of their genomes (1–7). One such recoding event is −1 programmed ribosomal frameshifting (−1PRF), where ribosomes slip back by one nucleotide in the 3′ to 5′ direction (−1) into an alternative reading frame. The frameshift stimulatory element (FSE) that promotes frameshifting typically consists of a slippery site (SS, the heptanucleotide UUUAAAC in SARS-CoV-2, Figure 1A) and an RNA structure in form of a stem loop or pseudoknot located at a defined distance of 5–9 nucleotides downstream of the SS (1,7–9). The role of the PK structure is to slow down translation elongation by impeding translocation of the tRNAs over the slippery codons and thereby facilitating new codon-anticodon interactions in the alternative reading frame (10,11).

Since the outbreak of the COVID-19 pandemic, several studies explored the structure of the SARS-CoV-2 FSE employing nuclear magnetic resonance (NMR), X-ray crystallography, cryo-electron microscopy (Cryo-EM), optical tweezers and chemical probing techniques (12–20). These structural studies of the PK RNA in solution or engaged with the ribosome show that it folds into an H-type pseudoknot with coaxially stacked SL1 and SL2 stems, which form a continuous helix, while SL3 is perpendicularly positioned with respect to this helix. Several groups have proposed that the 5′ of the RNA can pass through a ring formed inside the three stems of the PK—this particular PK fold was named the threaded PK (13,19). On the ribosome, this well-structured RNA directly interacts with the proteins of the small subunit positioned at the mRNA entry channel and thus creates a mechanical hindrance to resist unwinding by the intrinsic helicase activity of the ribosome (12). Recently published SARS-CoV-2 RNA structure probing data *in vitro* and in infected cells indicate that the frameshift element can also be found in alternative conformations (15,16,21,22). *In silico* work by Schlick *et al.* also predicted several alternative folds, including a three-way junction (3WJ) forming with the 3′ and 5′ immediate ends of the PK and an alternative PK where the SL1 loop base pairs with nucleotides upstream of the FSE (21,23,24). In some cases, sequences upstream or downstream of the core FSE were proposed to affect frameshifting. For example, in SARS-CoV, an attenuator hairpin upstream of the PK was suggested to moderate frameshifting (25–27). In infected cells, several groups identified the 5′ upstream attenuator hairpin as part of an extended stem of varying lengths, which they termed the alternative stem or alternative duplex (15,22). Despite the varying length, there is a consensus that the alternative stem would include the slippery sequence and the SL1 of the PK, making it unable to fold into the PK (15,22). Recently, a similar structure was also supported by *in situ* conformation sequencing of RNA inside SARS-CoV-2 particles (28). Lastly, longer-range interactions of the FSE were predicted in genome-wide studies; these might have functions in structural organization or replication of the genome (15,20). Collectively, these data indicate that, although the isolated FSE contains a pseudoknot as the dominant fold, as the length of the frameshift RNA increases the molecule can be found in multiple alternative conformations, which may have functional roles during frameshifting and viral replication (15,21).

To gain a comprehensive view of the conformational heterogeneity of the SARS-CoV-2 FSE, we used an integrative approach with single-molecule optical tweezers (OT) and chemical probing by dimethyl sulfate mutational profiling (DMS-MaP) (Figure 1B and C). To determine the functional relevance of the respective conformers, we employed a flow cytometry-based cellular frameshift reporter assay (Figure 1D). This allowed us to evaluate the functional effect of different RNA variants on translation from the −1 reading frame (29). We show that the canonical PK is the effective structure to stimulate frameshifting, and that both the standard and the threaded PK folds can induce frameshifting (13,19). Through employing 5′ and 3′ extended FSE constructs and mutants that interfere with the folding of the PK (Supplementary Figure S1), we demonstrate that 25–50 nucleotides at the 5′ and 3′ regions of the FSE promote alternative folds that interfere with the formation of the frameshift stimulatory PK fold and lead to differences in frameshifting efficiencies. Furthermore, we were able to modulate the folding of the FSE by occluding specific base-pair interactions involving SL2 and 3′ downstream regions by employing antisense oligonucleotides (ASOs). Using locked nucleic acid (LNA) containing ASOs targeting the SL2 of the pseudoknot we show that we can abolish frameshifting *in vitro* and a in cell-based reporter assay. Taken together, our dynamic- and steady-state analysis of the frameshift RNA suggest that there are alternative conformations of the FSE mediated by *cis*-acting short-range RNA interactions that fine-tune gene expression of SARS-CoV-2, which would provide important clues for future structure-based drug design studies.

## MATERIALS AND METHODS

### Plasmid construction

**Figure 2.**
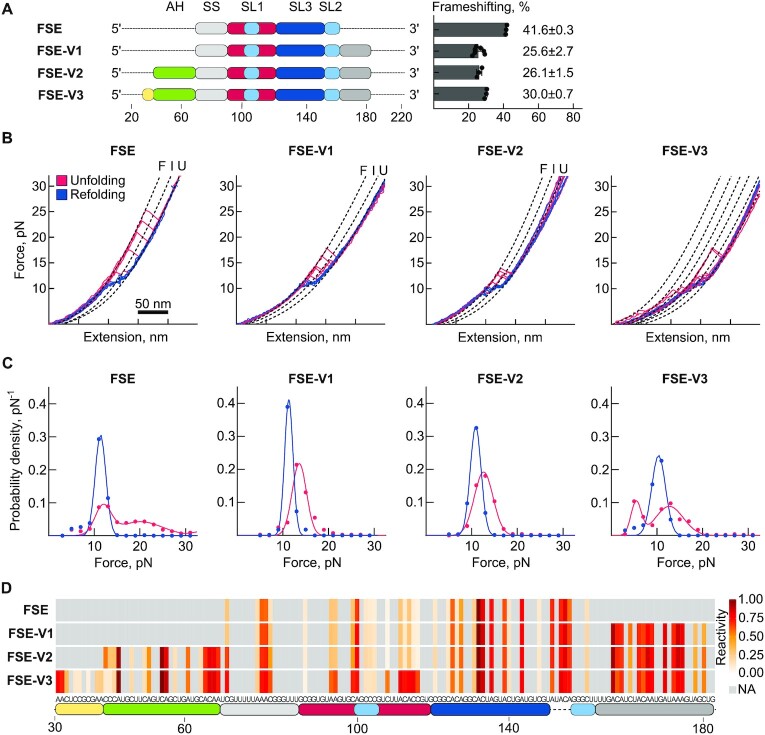
Effects of 5′ and 3′ extensions on frameshift efficiencies and RNA structure. (**A**) Schematic representation of RNA variants with respective parts of the FSE employed in the study. The color scheme matches the SARS-CoV-2 FSE in Figure 1. The yellow and green blocks correspond to the alternative stem 1 forming regions (AS1) and attenuator hairpin (AH), respectively. Numbers represent position relative to the beginning of the FSE-V4. Frameshift efficiencies of the variants are shown on the right. (**B**) Example unfolding (red) and refolding (blue) force-distance (FD) curves. ‘F’ denotes the folded states, ‘I’ the intermediate, and ‘U’ the fully unfolded state. (**C**) Histograms of the force distribution for the unfolding (red) and refolding (blue) steps observed in each sample. (**D**) Reactivity profiles of the RNA variants as determined by DMS-MaP.

To generate dual-fluorescence reporter constructs, frameshift sites of SARS-CoV-2 and corresponding mutant/truncated variants were placed between the coding sequences of EGFP and mCherry (parental construct was a gift from Andrea Musacchio (Addgene plasmid #87803 (30)) by site-directed mutagenesis or Golden Gate Assembly in a way that EGFP would be produced in 0 frame and mCherry in −1 frame. EGFP and mCherry were separated by StopGo signals (31) as well as an alpha-helical linker (32). A construct with no PRF insert and mCherry in-frame with EGFP served as a 100% translation control and was used to normalize EGFP and mCherry intensities. Variants and mutants of the frameshift site of the SARS-CoV-2 in the dual-fluorescence construct, as described in Figures 2 and 4 and Supplementary Table S1, were generated by Golden Gate Assembly.

OT constructs were based on the wild-type SARS-CoV-2 frameshift site (nucleotides 13475–13541) cloned into the plasmid pMZ_lambda_OT, which encodes for the handle sequences of lambda DNA (2 kb each) flanking the RNA structure of interest (29,33). Constructs were generated using Gibson Assembly. The additional RNA variants and mutants were generated by site-directed mutagenesis. Sequences of the variants used in this study are given in Table S1.

### Optical tweezers constructs

5′ and 3′ DNA handles, and the template for *in vitro* transcription of the RNA samples were generated by PCR using the pMZ_lambda_OT vector. The 3′ handle was labeled during the PCR using a 5′ digoxigenin-labeled reverse primer. The 5′ handle was labeled with biotin-16-dUTP at the 3′ end following PCR using T4 DNA polymerase. The RNA was *in vitro* transcribed using in-house purified T7 RNA polymerase. Next, DNA handles (5′ and 3′) and *in vitro* transcribed RNA were annealed in a mass ratio 1:1:1 (5 μg each) by incubation at 95°C for 10 min, 62°C for 2 h, 52°C for 2 h and slow cooling to 4°C in annealing buffer (80% formamide, 400 mM NaCl, 40 mM HEPES, pH 7.5 and 1 mM EDTA, pH 8) to yield the optical tweezer suitable construct. Following the annealing, samples were concentrated by ethanol precipitation, pellets were resuspended in 50 μl RNase-free water, and 4 μl aliquots were stored at −80°C until use.

### Optical tweezers data collection and analysis

Optical tweezers measurements were performed using a commercial dual-trap platform coupled with a microfluidics system (C-trap, LUMICKS) as described before (29,33,34). For the experiments, optical tweezers (OT) constructs were mixed with 4 μl of polystyrene beads coated with antibodies against digoxigenin (AD beads, 0.1% w/v suspension, Ø 2.12 μm, Spherotech), 10 μl of assay buffer (20 mM HEPES, pH 7.6, 300 mM KCl, 5 mM MgCl_2_, 5 mM DTT and 0.05% Tween 20) and 1 μl of RNase inhibitor (Molox). The mixture was incubated for 20 min at room temperature in a final volume of 19 μl and subsequently diluted by the addition of 0.5 ml assay buffer. Separately, 0.8 μl of streptavidin-coated polystyrene beads (SA beads, 1% w/v suspension, Ø 1.76 μm, Spherotech) were mixed with 1 ml of assay buffer. The flow cell was washed with the assay buffer, and suspensions of streptavidin beads and the complex of OT construct with anti-digoxigenin beads were introduced into the flow cell. During the experiment, single AD and SA beads were trapped in individual traps and brought into proximity to allow the formation of a tether. The beads were moved apart (unfolding) and back together (refolding) at a constant speed (0.05 μm/s) to yield the force-distance (FD) curves. The stiffness was maintained at 0.31 and 0.24 pN/nm for trap 1 (AD bead) and trap 2 (SA bead), respectively. FD data were recorded at a rate of 78125 Hz.

Raw data files were processed using the Practical Optical Tweezers Analysis TOol (POTATO, https://github.com/REMI-HIRI/POTATO (35)). In brief, raw data were first downsampled by a factor of 30 to speed up subsequent processing, and the noise was filtered using Butterworth filter (0.005 filtering frequency, filter order 4). Folded as well as unfolded regions of the FD curves were then fitted. For data fitting, we employed a combination of two worm-like chain models (WLC1 for the fully folded double-stranded parts and WLC2 for the unfolded single-stranded parts), as described previously (34). First, the initial contour length of the folded RNA was set to 1256 ± 5 nm, and the persistence length of the double-stranded part was fitted. Then, the persistence length of the unfolded RNA was set to 1 nm, and the contour length of the single-stranded part was fitted. The fitting parameters were derived and the results were plotted using Prism 9.2.0 (GraphPad).

### Cell culture

HEK293 cells (gift from Prof. Jörg Vogel, HIRI-HZI) were maintained in DMEM (Gibco) supplemented with 10% FBS (Gibco) and 100 μg/ml streptomycin and 100 U/ml penicillin at 37°C with 5% CO_2_. Transfections were performed using linear 25 kDa PEI (Polysciences) according to manufacturer's instructions using a 1:12 DNA:PEI ratio. For co-transfections, plasmids and antisense oligonucleotides were mixed in a 1:40 molar ratio and electroporations were performed in OptiMEM (Gibco) using a Nepa21 Super Electroporator (NEPAGENE) following the manufacturer's instructions. 24 h post-transfection, the cells were analyzed by flow cytometry. Antisense oligonucleotides (IDT) were added to the cells in a final concentration of 100 nM. To increase stability of the oligonucleotides, the phosphate backbone was substituted by phosphothioate and three bases at the 5′ and 3′ end were locked nucleic acids (LNAs).

### Flow cytometry

HEK293 cells were transiently transfected with either the control construct or the −1PRF construct encoding for the dual-fluorescence EGFP-mCherry translation reporter as outlined in Figure 1D. Cells were harvested at 24 h post-transfection. After washing with phosphate-buffered saline (PBS), flow cytometry was performed on a NovoCyte Quanteon (ACEA) instrument. FE was calculated according to the following formula:

$$\begin{equation*}{\rm{FE}}\left( {\rm{\% }} \right) = \frac{{\rm{mCherry}}_{{\rm{test}}}/{\rm EGF}_{\rm test}}{{\rm mCherry}{_{\rm control}}/{\rm EGFP}{_{\rm control}}}\times 100\end{equation*}$$

where mCherry represents the mean −1-frame mCherry intensity, EGFP the mean EGFP intensity, test represents the sample containing the frameshift element and control represents the in-frame control lacking the frameshift element where mCherry and EGFP are produced from the 0-reading frame. To ensure reliability of the present assay, several controls were employed. These included vectors with deleted CMV promoter, deleted slippery sequence, mutated slippery sequence, and a readthrough control (Supplementary Figure S2). Data represents the results of at least three independent experiments. Data was analyzed and plotted in GraphPad Prism (version 9.2.0). For statistical analysis of FE in the presence of antisense oligonucleotides, an ordinary one-sided ANOVA was followed by a Brown-Forsythe test to ensure equal variance among the samples. Finally, a Dunnett's multiple comparisons test was employed to compare test samples to control constructs (non-targeting antisense oligonucleotides).

### *In vitro* translation in rabbit reticulocyte lysate (RRL)

*In vitro* translations in rabbit reticulocyte lysate (RRL) were performed as described before (29). Briefly, mRNAs were synthesized by *in vitro* transcription with in-house purified T7 polymerase using linearized plasmid DNAs as templates and subsequently translated using rabbit reticulocyte lysate (RRL; Promega). Typical reactions were 0.05 μM template mRNA, 75% v/v RRL, and 20 μM amino acids. Antisense oligonucleotides (IDT) were added to the reactions in a concentration range from 0–0.5 μM. Reactions were incubated for 1 h at 30°C at which point samples were mixed with three volumes of 1× BOLT™ LDS Sample Buffer (Invitrogen), denatured for 10 min at 70°C, and resolved on NuPAGE™ 4–12% Bis-Tris polyacrylamide gels (Invitrogen). Translation products were detected by western blot. Briefly, after transfer using Trans-Blot (Bio-Rad), nitrocellulose membranes were developed with anti-DYKDDDK-tag antibody (Proteintech 20543-1-AP, 1:3000) and visualized by incubation with a secondary antibody (IRDye^®^ 800CW Goat anti-rabbit, LI-COR, dilution 1:10000)). Bands were detected using an Odyssey Clx infrared imager system (LI-COR) and quantified densitometrically using FIJI software (36). FE was calculated as previously described, by the formula:

$$\begin{equation*}{\rm{FE}}\left( {\rm{\% }} \right){\rm{\;}} = \frac{{{\rm{Intensit}}{{\rm{y}}_{ - 1 - {\rm{frame}}}}}}{{{\rm{Intensit}}{{\rm{y}}_{0 - {\rm{frame}}}} + {\rm{Intensit}}{{\rm{y}}_{ - 1 - {\rm{frame}}}}}}\; \times 100\end{equation*}$$

The relative FE was calculated as a ratio of FE of each condition to the FE of no-oligonucleotide control in each measurement. Experiments were repeated at least three independent times. Data was plotted and IC50 values were calculated using an inhibitor vs. response model assuming a standard slope (Hill slope = −1) in GraphPad Prism (version 9.2.0) software.

### Dimethyl sulfate mutational profiling (DMS-MaP)

A mix of 37 nM of RNA, 37 nM of blocking primer 5′ [GTAGCTGTCGAGCTCCTGCGAAG] and 37 nM of blocking primer 3′ [GGCGAAGAGCAGGTTGCAGGAT] was first heat denatured at 90°C for 2 min, then annealed by slow cooling (<1°C/min) to 50°C. RNA was then folded by adding the folding buffer (20 mM HEPES, pH 7.6, 250 mM KCl, 5 mM MgCl_2_ and 0.3 U/μl of RNasin) and slow cooling (<1°C/min) to 23°C. DMS was diluted in ethanol to a working concentration of 0.85 M. 1/10 volume of DMS working stock was added to the samples to make a final concentration of 85 mM in a total volume of 33 μl. Samples were incubated at 37°C for 6 min and then quenched with 33 μl of beta-mercaptoethanol (14.2 M stock). For the untreated control, ethanol was used instead of DMS. RNA was then purified by Trizol LS (Sigma) according to the manufacturer's instructions.

Half of the RNA samples were reverse transcribed using 40 U MarathonRT (37,38) in RT Buffer (50 mM Tris–HCl, pH 8.3, 200 mM KCl, 5 mM DTT, 20% glycerol, 1 mM MnCl2), 0.5 mM dNTP mix, 0.3 μM primer [GGCGAAGAGCAGGTTGCAGGAT] and 8 U of RNasin in a final volume of 25 μl. Reverse transcriptions were carried out at 42°C for 4 h. cDNA was diluted 1/8 with nuclease-free water and PCR amplified. Reaction conditions were 8 μl of diluted cDNA, 1× GXL reaction buffer, 0.2 mM dNTPs, 0.25 μM of forward [TCGTCGGCAGCGTCAGCTTCGCAGGAGCTCGACAGCTAC] and reverse primer [GTCTCGTGGGCTCGGAGGGCGAAGAGCAGGTTGCAGGAT], 0.02 U/μl of Q5 High-Fidelity DNA Polymerase (NEB) in a final volume of 50 μl. Cycling conditions were 2 min at 98°C then 25 cycles of 10 s at 98°C, 15 s at 60°C and 15 s at 72°C then 72°C for 5 min. PCR products were verified on a 2% agarose gel followed by column purification (NucleoSpin Gel and PCR Clean-up kits, Macherey-Nagel) according to the manufacturer's instructions. A final indexing PCR was carried out using Illumina Nextera DNA CD indexes (96 Indexes, 96 Samples, Illumina). Reaction conditions were 40 ng of purified PCR product, 1 × Q5 reaction buffer, 0.2 mM dNTPs, 2.5 μl of indexing primer, 0.02 U/μl of Q5 polymerase in a final volume of 14 μl. Indexed PCR products were verified on a 1.5% agarose gel, pooled together in an equimolar ratio, before final purification on a 1.5% agarose gel. The pooled indexed sequencing library was quantified using the NEBNext library Quant Kit for Illumina and paired-end PE150 sequencing was carried out on an Illumina Novaseq instrument (Novogene).

DMS-MaP data was trimmed using cutadapt v 1.18 (39) and aligned to the reference sequence using bowtie2 (40). cutadapt parameters were ‘–nextseq-trim 20 –max-n 0 -a atcctgcaacctgctcttcgcc -A gtagctgtcgagctcctgcgaag’. bowtie2 parameters were ‘-D 20 -R 3 -N 1 -L 15 -i S,1,0.50’. Further analysis was carried out using the rf-count and rf-norm modules of RNA Framework package v2.7.2 (41). rf-count parameters were ‘-m -es’. rf-norm parameters were ‘-rb AC -sm 3 -nm 1’, meaning that DMS reactivities were calculated by subtracting background mutations in the untreated sample and normalized using 2–8% normalization (42). Alternative structures were detected directly from the DMS data using the clustering algorithm Detection of RNA folding Ensembles using Expectation-Maximization' (DREEM) (43). Briefly, RNA molecules from DMS-MaP experiments often contain multiple modifications that can be used later to distinguish between alternative folds. The DREEM algorithm uses expectation maximization to directly assign individual sequencing reads to distinct structural clusters. The number of clusters and their relative proportions are computed by iteratively maximizing a log-likelihood function based on a multivariate Bernoulli mixture model. The resulting DMS reactivities for each structural class were then used to predict RNA structures using RNAstructure using the default parameters embedded in the DREEM pipeline. Data were plotted using StructureEditor (version 1.0) (44).

## RESULTS

### 5′ and 3′ extensions of the FSE can favor alternative conformers

Frameshifting has been shown to be induced by a 68 nucleotide (nt) long pseudoknot structure within the FSE, yet analyses of the SARS-CoV-2 whole genome structure suggest that the region including the FSE can adopt multiple conformations in cells (15–17,21). In order to determine the translation relevant structural and functional effects proximal and distal nucleotides of the FSE, we designed and tested four variants (Figure 2A, Supplementary Figure S1). The reference RNA, which we call the core FSE of 86 nt, contains the slippery sequence, spacer and the pseudoknot composed of SL1, SL2 and SL3. FSE-V1 contains the FSE and an extended 3′ region of 28 nucleotides (total length: 114 nt). FSE-V2 contains extensions immediately 5′ and 3′ of the FSE, including the 5′ attenuator hairpin (length: 141 nt). FSE-V3 contains the 3′ extension (28 nt) and a 38 nt extension at the 5′ end, including the recently proposed alternative stem 1 (length: 152 nt) (15,22). Lastly, FSE-V4 contains the longest 5′ extension of 68 nucleotides and an additional 3′ extension of 39 nt (length: 221 nt) (Supplementary Figure S2A).

We first asked whether upstream and downstream regions of the FSE can alter frameshifting. For that we used a cell-based dual-fluorescence frameshift reporter, in which the SARS-CoV-2 frameshift site is placed between the EGFP gene in the 0-frame and the mCherry gene in the –1-frame (Figure 1D) (29). With the core FSE construct, frameshifting efficiency was 41.6 ± 0.3%. The presence of extensions on either 3′ (FSE-V1) or 5′ (FSE-V2) of the FSE resulted in a decrease of frameshifting levels to approximately 25% (Figure 2A). The FSE-V3 construct containing an additional 5′ extension had a frameshifting efficiency of 30% (Figure 2A) The FSE-V4 construct, which has the longest extension among the variants tested had the highest frameshift efficiency of about 37%, close to the stimulation observed with the wild type FSE (Supplementary Figure S3A).

In order to test whether the effect on frameshifting is related to altered conformation of the FSE variants, we next employed single-molecule optical tweezers (Figure 2B and C, Supplementary Tables S2 and S3). Here, FSE RNA variants flanked by 2 kb DNA:RNA hybrid handles were gradually stretched at a constant rate and then the applied force was released allowing the RNA molecule to refold. This allows the RNA molecule to shift between folded and unfolded states and sudden changes in measured force-distance (FD) trajectories represent transitions between RNA conformations (29) (Figure 1B). The (un)folding force as well as the change in contour length and folding hysteresis (the difference between the observed unfolding and refolding transitions) provides the information on RNA conformational states.

In the FSE RNA, which in addition to the PK contains the slippery sequence and the spacer, we mostly observed a single unfolding step in 72.2% of the FD trajectories (Figure 2B and C, Supplementary Table S3). Most of these unfolding events occurred at forces above 15 pN and exhibited hysteresis, which is typical for structured RNAs, like pseudoknots (19,45–48) (Figure 2B and C). In 20.8% of the FD curves, we noted two successive unfolding events (Table S3), each with an average of 17.1 ± 3.5 nm change in the contour length, which would correspond to the opening of ≈32 nucleotides. The total change in contour length of the FSE sample was 35.5 ± 2.5 nm, which was close to the expected length of 37.2 nm for the PK (68 nt) (13,19) (Supplementary Figure S4A and Supplementary Table S2). The two-step unfolding pattern may represent the sequential unfolding of individual stem-loops, SL2 + 3 and SL1 or may indicate presence of an alternative fold (Figure 2B and Supplementary Table S2). The histogram of the unfolding forces also pointed to the presence of at least two populations in the FSE sample (Figure 2C and Supplementary Table S2). First, one unfolding event at the force of 11.9 ± 1.8 pN, and a second event with a mean force of unfolding at 20.1 ± 4.9 pN (Table S2). Based on the individual refolding trajectories, the population unfolding at lower forces would correspond to a mixture of stem-loops and a less-stable PK fold (Figure 2B and C). The second population unfolding at higher forces corresponds to a highly stable PK fold, which likely represents the threaded PK (13,19) (Figure 2B and C).

Previous studies also predicted that the 3′ end of the PK can be involved in alternative folds, with unknown functions (15,16,20–22). To test whether the immediate 3′ nucleotides lead to the formation of conformations that compete or co-exist with the FSE and affect the dynamics of the canonical FSE, we employed the FSE-V1 variant (Figure 2A and Supplementary Figure S1). With this RNA variant we noticed a decrease in single-step unfolding events from 72% to 55% as compared to the FSE sample (Table S3). In addition, we marked a decrease in the population of a highly stable conformer, which we infer to be the threaded PK, although the less stable PK fold was still pronounced (Figure 2B, C and Supplementary Table S2). The total change in contour length of the FSE-V1 sample was slightly increased (37.5 ± 3.6 nm) compared to FSE (Supplementary Figure S4B and Supplementary Table S2).

Next, we employed the FSE-V2 RNA variant containing the putative attenuator hairpin at the 5′ of the FSE, which was previously reported to decrease frameshifting in SARS-CoV-1 and SARS-CoV-2 (25,26) (Figure 2A and Supplementary Figure S1). The (un)folding behavior of the FSE-V2 variant was similar to the FSE-V1 sample, with no noticeable threaded PK-like unfolding events and a similar change in the contour length (Figure 2B, C, Supplementary Figure S4C and Table S2). However, we see an increase in two step unfolding event (Table S3). Interestingly, during unfolding of both RNA variants with 5′ and/or 3′ extensions we also observed a few folding rescue events (Supplementary Figure S5). These events were characterized by reversible folding behavior during pulling, suggesting that the RNA molecule dynamically explores various intermediate states, while it partially unfolds and immediately refolds into an energetically more favored structure (49).

We then evaluated the effect of the alternative stem (AS) (or the so-called alternative duplex) formed by base pairing between the 5′ upstream region of the attenuator hairpin and parts of the FSE including the stem of SL1 (15,22). For that, we designed the FSE-V3 variant containing an 11 nt extension 5′ of the attenuator hairpin (Figure 2A and Supplementary Figure S1). Similar to the FSE-V1 and FSE-V2 variants, the threaded PK state was absent in the FSE-V3 sample (Figure 2B and C). Unlike the other variants, we noted a substantial population of unfolding steps at lower forces (5.4 ± 1.1 pN) and overall higher heterogeneity of FD trajectories, pointing to the presence of less stable short hairpins in the RNA (Figure 2B, C, Supplementary Figures S4D, S5, and Supplementary Tables S2 and S3). Lastly, we tested the effect of further extending the 5′ end of the FSE by 68 nucleotides (FSE-V4) (Supplementary Figures S1 and S3). This sample was found to be highly heterogeneous indicating presence of at least 3 low force unfolding hairpins, together with a higher force unfolding population with a large change in the contour length by 102.0 ± 10 nm (Supplementary Figure S3 and Supplementary Table S2).

Overall, based on our single-molecule analysis we conclude that the PK is the major conformation for the FSE, yet the FSE can adopt pseudoknot structures with varying stabilities. Interestingly, addition of 5′ and 3′ proximal nucleotides to the canonical FSE resulted in the loss of the highly stable RNA structures, suggestive of alternative no-PK folds present together with a low stability PK.

### Chemical probing of the structural ensembles confirms presence of alternative folds

To investigate the conformational heterogeneity of the FSE and its variants, we next generated structural profiles of the RNAs based on reactivities of RNA to the chemical DMS (50,51) (Figure 1C). In the FSE sample, DMS reactivities were consistent with the formation of the pseudoknot, especially the nucleotides within the loop of SL1 involved in the formation of SL2 were unreactive to DMS (Figure 2D). Importantly, SL1 loop nucleotides became reactive to DMS in constructs containing deletions in the 3′ stem of SL2, together with a deletion of SL3 (FSE-D1) or alone (FSE-D2) (Supplementary Figure S6). However, in the absence of the SL3 alone (FSE-D3), SL1 loop nucleotides remained unreactive and indicating the formation of a minimal PK composed of SL1 and SL2, which was still able to support frameshifting in a reporter assay, in contrast to the FSE-D1 and FSE-D2 mutants which were unable to frameshift (Supplementary Figure S6).

In the FSE-V1 and FSE-V2 samples we saw negligible differences compared to the FSE sample (Figure 2D and Supplementary Figure S7). In the FSE-V3, which contained the putative alternative stem at its 5′ end, more prominent changes in DMS reactivities were seen (Figure 2D and Supplementary Figure S7). The most striking change was the strongly increased reactivity in both strands of the SL1 stem, particularly nucleotides 110–114, which were protected in the FSE sample and other FSE RNA variants (Figure 2D and Supplementary Figure S7), indicating structural rearrangements of the FSE consistent with the unstable folding states observed in single-molecule pulling experiments (Figure 2B and C). FSE-V4 showed increased DMS reactivities within SL1 suggestive of an alternative fold similar to FSE-V3 (Supplementary Figures S3E and S7).

**Figure 3.**
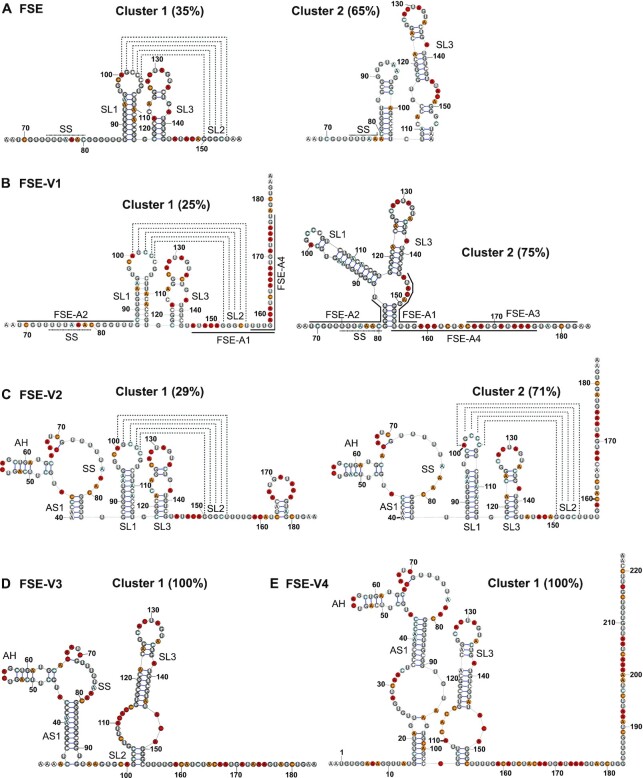
DMS-guided cluster analysis of FSE variants. (A–E) Secondary structures detected based on clustering of DMS reactivities using DREEM. DREEM does not predict pseudoknots, and dotted lines represent PK interactions supported by DMS-MaP. Structural elements are annotated according to the nomenclature described in the manuscript (**A**) FSE, (**B**) FSE-V1, binding sites of oligonucleotides used in this study are marked, (**C**) FSE-V2, (**D**) FSE-V3, (**E**) FSE-V4.

To probe for alternative folds, we next analyzed DMS-MaP data at the single-molecule level using the Detection of RNA folding Ensembles using Expectation-Maximization' (DREEM) algorithm (15). DREEM clusters single-molecule measurements of RNA structure in DMS-MaP experiments to detect and quantify the relative abundance of alternative structures. In the FSE sample, we detected two alternative conformations (Figure 3A). Cluster 1 was detected at 35% abundance and is consistent with the canonical PK structure. Cluster 2 was detected at 65% abundance and contained a shifted SL1 structure, which no longer exposed the loop required for PK formation (Figure 3A). In FSE-V1, we identified two conformations, with a decrease in the relative abundance of the putative PK structure from 35% to 25% (Figure 3B). The second conformation was predicted to form a 3WJ, consistent with the one proposed by Schlick *et al.* (21,24). Interestingly, formation of this 3WJ conformer is further supported by DMS reactivities at U residues, which were recently demonstrated to be also susceptible to DMS (52) (Supplementary Figure S7). U bases in the immediate upstream (84–86 nt) and downstream (159–160 nt) of the PK, which would be base-paired in the 3WJ fold, show substantially lower reactivity compared to the same bases in FSE sample (Supplementary Figure S7). In FSE-V2, two very similar clusters were detected at relative proportions of 29% and 71% (Figure 3C). The most notable difference in DMS reactivities between these two clusters was seen at a single position (C99) in the loop of SL1, suggestive of a subtle change in the PK structure (Figure 3C). In FSE-V3, intriguingly, we detected a single structural cluster which maintained the canonical SL3 and SL2 structures. However, SS and SL1 were folded differently as part of the alternative stem structure (AS1) (Figure 3D). Finally, FSE-V4 was seen as a single structural cluster, containing a slightly expanded SL3 and a long alternative stem (AS1) similar to the ones reported in chemical probing experiments conducted in cells and virions (15,22,28) (Figure 3E).

### Mutations of FSE can favor alternative folds

**Figure 4.**
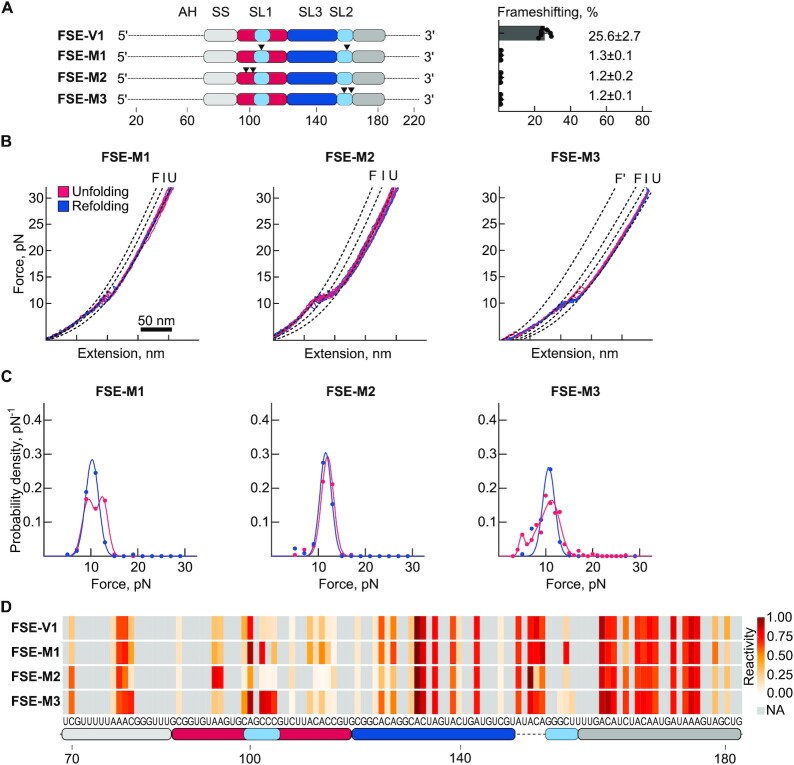
Effect of point mutations altering the conformation of the FSE. (**A**) Schematic representation of RNA mutants. Frameshift efficiencies of each RNA as measured by dual-fluorescence assay are plotted on the right. (**B**) Example unfolding (red) and refolding (blue) force-distance (FD) curves. ‘F’ and ‘F'’ denote different folded states, ‘I’ the intermediate state, and ‘U’ the fully unfolded state. (**C**) Histograms of force distribution for the unfolding (red) and refolding (blue) events observed in each RNA sample. (**D**) Reactivity profiles of the RNA mutants as determined by DMS-MaP.

To explore the role of the PK structure and to dissect how alternative conformations of the FSE might impact frameshifting, we designed FSE mutants (FSE-M1-3), based on the FSE-V1 mRNA. These mutants were computationally predicted to either disrupt the PK fold or alter equilibrium between the alternative folds identified above (24) (Figure 4A and Supplementary Figure S1).

FSE-M1 was designed to interfere with PK formation by disrupting SL2 whilst leaving SL1 and SL3 of the FSE intact (Figure 4A and Supplementary Figure S1) (24). Most of the FD curves (80.7%) presented a two-step unfolding pattern, first a small step at 9.4 ± 1.3 pN followed by a second step at 12.6 ± 1.1 pN with total contour length change of 26.1 ± 3.4 nm (Figure 4B, C, Supplementary Figure S4E, and Supplementary Tables S2 and S3). Furthermore, the pattern of unfolding in the FSE-M1 mutant showed remarkable similarity to the FSE-D2, which lacks the 3′ strand of SL2, indicating that these two steps correspond to opening of the SL3 and SL1, respectively (29). In the remaining 20% of the FD curves, we detected only the second step around 13 pN, denoting that the SL3 either did not form or the transition was below the detection limit (Table S2). In addition, DMS-MaP data pointed to increased reactivities in SL2 indicating that this mutant disrupted the pseudoknot structure (Figure 4D). Frameshifting efficiencies dropped from 25.6% to 1.3% demonstrating that SL1 and SL3 are not sufficient for frameshifting (Figure 4A).

The FSE-M2, contains two point mutations at the 3′ end of SL1 (Figure 4A and Supplementary Figure S1). This mutant is predicted to shift the location of SL1 by forming additional base pairing with the spacer and the C of the slippery sequence (UUUAAAC), forming a structure similar to cluster 2 of FSE (Figure 3A) (24). In pulling experiments, FSE-M2 unfolded in two steps in most of the FD curves (69.5%) both at 11.9 ± 1.3 pN with total contour length change of 36.3 ± 1.6 nm (Figure 4B-C, Supplementary Figure S4F and Supplementary Tables S2 and S3). DMS-MaP data indicated an increase in DMS reactivity of two A residues at positions 94–95 found at the 5′ strand of SL1, supporting the formation of a shifted SL1 structure (Figure 3A, cluster 2 and Figure 4D). In cell-based reporter assays, frameshifting efficiencies dropped to 1.2% demonstrating that cluster 2 as seen in the FSE sample does not induce frameshifting. This further reinforces the notion that cluster 1 detected in the FSE sample is the frameshift stimulatory fold (Figure 3A).

The FSE-M3 RNA variant has two mutations in the 3′ strand of SL2 (Figure 4A and Supplementary Figure S1). These mutations are designed to stabilize base pairing interactions between the 5′ spacer and the 3′ strand of SL2, thus promoting the folding of the 3WJ as detected in cluster 2 of FSE-V1 (Figure 3B) (24). The majority of unfolding events occurred in three steps (4.8 ± 0.5 pN, 8.0 ± 2.1 pN, and 11.3 ± 1.8 pN) accompanied with similar low force refolding with a total contour length of 69.7 ± 3 nm, corresponding to the full unfolding of all 114 nt (Figure 4B, C, Supplementary Figure S4G and Supplementary Tables S2–S3). The unfolding step at low forces (4.8 ± 0.5 pN) is most likely the opening of the base of the 3WJ (Table S2). As expected, DMS-MaP data showed asymmetric changes in DMS reactivity at SL2, with increases in reactivity of the 5′ strand of SL2, but the 3′ strand of SL2 remaining unreactive (Figure 4D and Supplementary Figure S7). As seen with the FSE-M1 and FSE-M2 mutants, frameshifting efficiencies dropped to 1.0% demonstrating that the 3WJ is not able to promote frameshifting (Figure 4A).

Another FSE mutant contained two substitutions in SL2 (FSE-M4), which was predicted to form an alternative PK structure (Supplementary Figures S8A and S1) (24). However, our single molecule OT analysis did not indicate any PK-like unfolding trajectories (Supplementary Figure S8B–D). DMS-MaP showed increased reactivities in SL2, but we did not observe any reactivity differences in SL1 and SL3 suggesting that these mutations result in the disruption of the PK fold, leaving SL1 and SL3 intact (Supplementary Figure S8E). In line with that, FSE-M4 was not able to frameshift efficiently (1%) (Supplementary Figure S8A).

Thus, we conclude that the correct folding of the pseudoknot is crucial for frameshifting and that alternative folds, such as the 3WJ, can compete with PK formation to decrease frameshifting efficiency.

### Antisense oligonucleotides can alter FSE conformations

**Figure 5.**
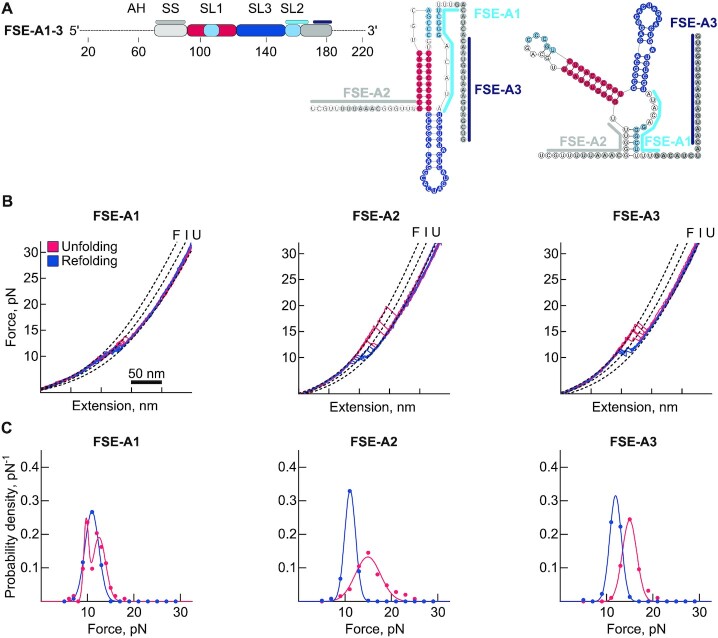
Antisense oligonucleotides alter the formation of conformers. (**A**) A schematic representation of the ASO samples. The oligonucleotide binding sites on the RNAs are depicted on the PK and 3WJ conformers. (**B**) Example unfolding (red) and refolding (blue) force-distance (FD) curves. ‘F’ denotes the folded, ‘I’ the intermediate, and ‘U’ the fully unfolded states. (**C**) Histograms of force distribution of the unfolding (red) and refolding (blue) steps observed in each sample.

Informed by our analysis of the FSE structural folds, we next tested three antisense oligonucleotides (ASO) designed to alter the balance between PK and alternative folds (Figure 5A and Supplementary Figure S1).

The first ASO (FSE-A1) hybridizes to the 3′ strand of SL2 (position 145–158 relative to the FSE-V4) and thus impairs folding of the canonical PK (Figures 5A and 3B cluster 1). Here, we expected to observe (un)folding behavior comparable to the FSE-D2 and FSE-M1 samples, in which the PK forming SL2 was either deleted or mutated (Figure 4B, C and Supplementary Figure S4E) (29). In agreement, in the presence of the FSE-A1 oligonucleotide about 90% of the (un)folding events occurred in two steps, at forces of 9.4 ± 1.3 pN and 12.6 ± 1.1 pN, showing a shift to low-stability hairpins (Table S2). Furthermore, the total change in contour length of 32.7 ± 5.1 nm was similar to the contour length change we observed with the FSE-M1 and FSE-D2 samples which cannot form SL2 (Figure 4, Supplementary Figures S4E, 4H and Supplementary Table S2) (29). Based on these data, we conclude that the unfolding profile of FSE-A1 represents the sequential opening of SL1 and SL3, indicating the successful disruption of the canonical PK (Figure 5B, C and Supplementary Tables S2-S3).

The second ASO (FSE-A2) binds to the 5′ end of the PK including the SS and the spacer region (position 69–86), which is a few nucleotides downstream of the attenuator stem (Figure 5A and Supplementary Figure S1). This ASO mimics the ribosome induced unfolding of the RNA 5′ to the PK. This means that it would not disrupt folding of the PK directly but may influence alternative folds, for example by preventing base pairing at the base of the 3WJ. In this sample, we noted an increase in single-step unfolding events (from 55.5% in FSE-V1 to 82.7%), and the average force of unfolding was slightly increased to 14.9 ± 2.7 pN, which implies the presence of more PK fold in this sample (Figure 5B, C, Supplementary Figure S4I and Supplementary Tables S2 and S3). However, we did not observe the highly stable PK fold, presumably because the 5′ immediate region cannot fold into the threaded form once it is hybridized to the ASO (Figure 5B and C).

Lastly, we tested FSE-A3, which binds 10 nucleotides downstream of the FSE (position 166–182), a region that was not predicted to be part of any of the major conformers (Figure 5A). Accordingly, the unfolding profile and the length of the unfolded region in single-molecule experiments were mostly unchanged compared to the FSE-V1 variant (Figure 5B, C, and Supplementary Tables S2-S3). In this sample, we observed a small increase in unfolding force (15.0 ± 1.5 pN) and the number of single-step unfolding events as compared to FSE-V1 (65.2%) (Figure 5B, C and Supplementary Figure S4J, and Tables S2–S3).

Overall, these data show that a preclusion of the base pairing of 5′ spacer region and the 3′ strand of the SL2 hampers the formation of the alternative folds, further suggesting that FSE can undergo structural rearrangements during translation, when the slippery nucleotides and the spacer region are occluded by the ribosome.

### Antisense oligonucleotides can alter frameshifting efficiencies

To test whether ASOs can affect frameshifting, we employed in cell dual-fluorescence reporter assays and *in vitro* translation reporter assays (53,54). This strategy has been successfully employed to target untranslated regions (UTRs) and FSEs in SARS-CoV-2 and other viruses (13,55–58).

**Figure 6.**
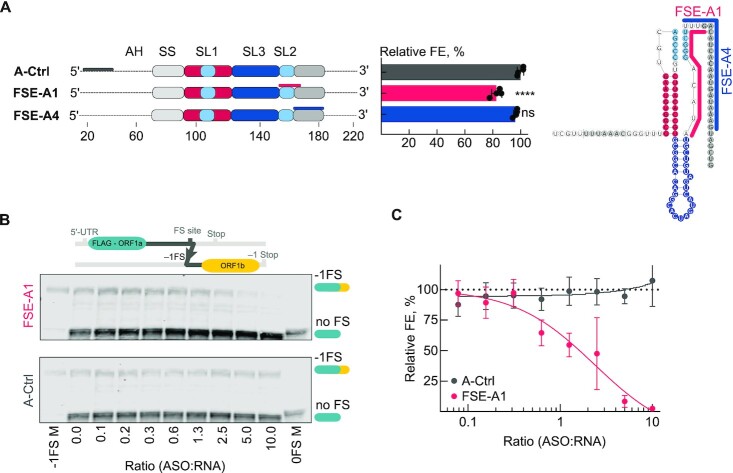
Targeting the FSE with antisense oligonucleotides. (**A**) Schematic representation of the binding sites of the ASO employed in the study. *In vivo* dual-fluorescence assay to evaluate the effect of ASOs on PRF in HEK293 cells. The relative frameshifting efficiency (FE) is calculated from the ratio of mCherry to EGFP intensities in the frameshift construct relative to the in-frame control construct lacking the PRF signal. Datapoints represent the mean ± s.d. (*n* = 4 independent experiments). *P*-values were derived by an ordinary unpaired one-sided ANOVA comparing every ASO to the scrambled FSE-A-Ctrl for each construct. *P < 0.05, ****P < 0.0001. (**B**) Upper panel: Scheme of the SARS-CoV-2 mRNA construct used for *in vitro* translation experiments. The N-terminal FLAG-tagged frameshifting reporter construct includes the nucleotides 12686–14190 (≈1.5 kb) of the SARS-CoV-2 genome. Representative western blots ASO titrations in RRL-based *in vitro* translation using the FSE-A1 (upper) or the control (A-Ctrl) oligonucleotide. Frameshift constructs encode for an N-terminal FLAG-tagged version of the 1a-1b polyprotein. 0-frame frameshift product: 32 kDa, −1-frame product: 58 kDa. (**C**) Graph presenting the densitometric quantification of the western blot analysis in (**B**). Datapoints represent the mean ± s.d. (n = 3 independent experiments); IC50 (FSE-A1) = 1.8; *R*^2^ = 0.94. For additional controls, please refer to Figure S2.

We first tested ASO FSE-A1, which impairs the formation of the PK (Figure 6A). Accordingly, we observed a 20% decrease in frameshifting in the cell-based reporter assay (Figure 6A). To probe for dose dependency of the effect observed with the FSE-A1 sample, we next used an *in vitro* frameshift reporter assay (Figure 6B). In contrast to the cellular reporter assay, where we employed isolated frameshift site fragments, here the frameshift construct was derived from the SARS-CoV-2 genomic RNA corresponding to a 1.5 kb long region (nucleotides 12686–14190) of the ORF1a/1b (29). Therefore, the *in vitro* SARS-CoV-2 frameshift reporter also enabled testing whether *cis*-RNA interactions within the 1.5 kB long region of SARS-CoV-2 affect frameshifting, albeit not excluding the possibility of other long-range base pairing of the FSE beyond this region. Translation of the *in vitro* frameshift reporter RNA results in a 31.8 kDa long peptide in the 0-reading frame, and a longer product of 59.2 kDa in the −1 frame (29). We observed a strong dose dependent decrease in frameshifting, ending up in complete inhibition of frameshifting at the 10:1 ratio (ASO:RNA) (Figure 6B and C). On the other hand, with the non-targeting control oligonucleotide, we did not observe a difference in the relative amounts of the −1-, or 0-frame products. The difference in the targeting efficiency of FSE-A1 ASOs in cell-based versus *in vitro* reporter assays can be attributed to low transfection efficiencies, which may limit the cellular levels of ASOs to suboptimal concentrations.

Next, we aimed to test whether we could alter frameshifting efficiencies by targeting alternative conformers of the FSE. Since FSE-A2 hybridizes with the SS, which would interfere with ribosome progression and translation independent of frameshifting, we designed FSE-A4 to interfere with the formation and stability of alternative folds involving 3′ downstream regions of the PK. The 3WJ conformer as detected by the DREEM analysis would be only partially targeted with this ASO, nevertheless one can expect some destabilization of the 3WJ (Figure 6A). Yet, we detected no significant effect on frameshifting in the presence of the FSE-A4 (Figure 6B).

To sum, our *in vitro* and cell-based reporter assays further corroborate that the PK is the critical fold for efficient frameshifting and can be effectively targeted by antisense strategies.

## DISCUSSION

Due to its crucial role in the viral life cycle and replication, the FSE of SARS-CoV-2 has been extensively studied by functional and structural approaches (12,13,15–19,21,22,28). These studies imply that FSE folding varies substantially depending on whether the FSE RNA is investigated in isolation or in the presence of 5′ and 3′ extensions that more closely mimic the genomic context. As a result, there is a lack of consensus on the existence of alternative conformations of the FSE, and their functional relevance to frameshifting.

**Figure 7.**
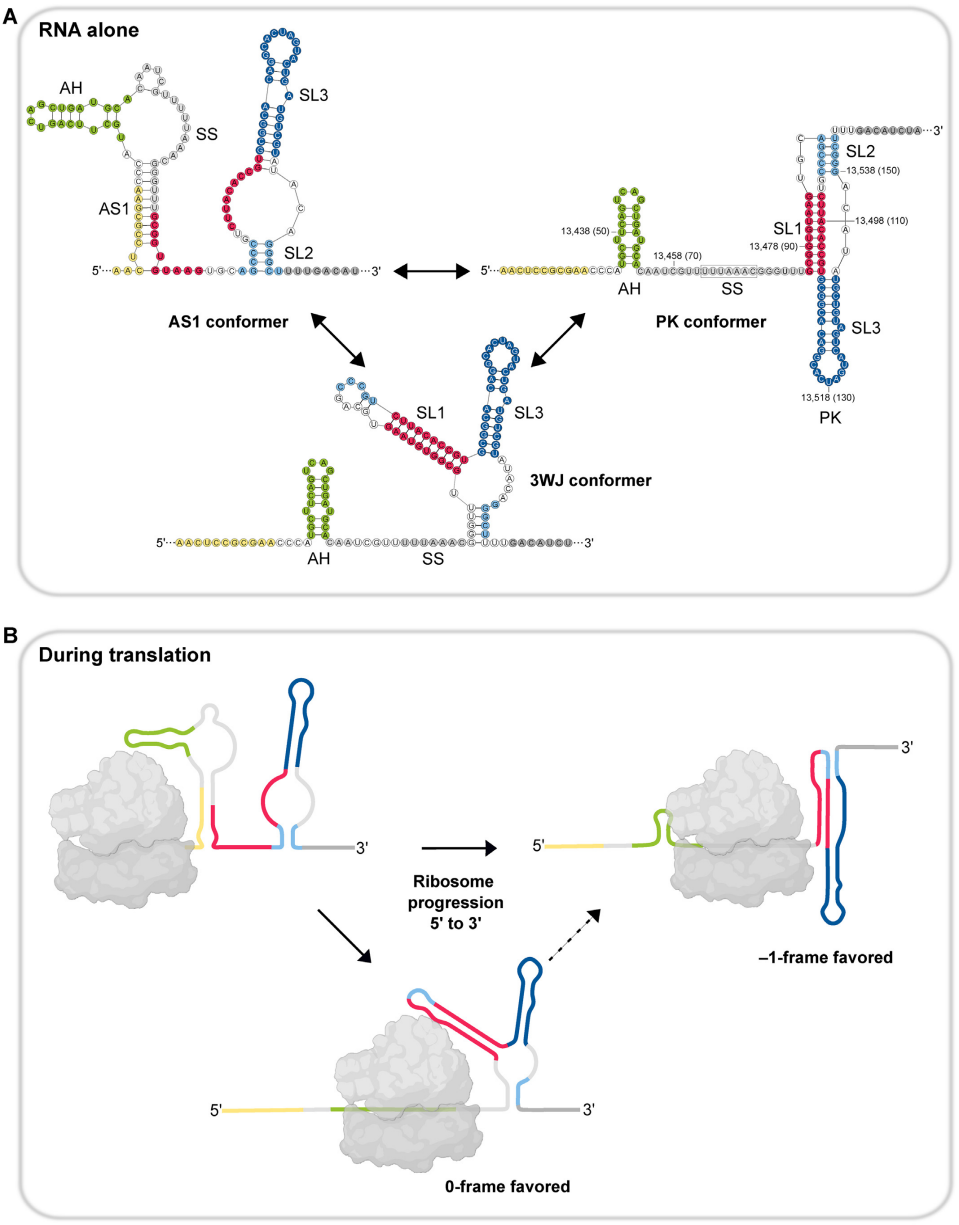
Proposed model of PRF site conformational transitions as a translation regulatory switch. (**A**) Transition between the alternative conformation, the canonical pseudoknot and the three-way junction is achieved by a conformational switch between SL1, AS1 and SL2. (**B**) In cells, the viral RNA undergoes translation, replication/transcription or virion packaging depending on its localization and phase of infection. The viral RNA is trapped in an intermediate conformation. During translation, as the ribosome progresses, the AS1 of the intermediate conformer would be unwound by the helicase activity of the ribosome, thus allowing SL1 to form. This could result in formation of either the frameshift stimulatory PK or the three-way junction. As translation progresses, the three-way junction may also fold into the PK.

Here, we used single molecule optical tweezers and DMS-MaP structural probing to investigate the folding and unfolding dynamics and steady-state RNA conformers of SARS-CoV-2 FSE variants. Collectively, our results show that the PK is not the only conformer formed at the frameshift site. Instead, immediate 5′ upstream and 3′ downstream regions of the FSE promote structural transitions of the FSE. In the presence of these regions, the frameshift stimulatory PK fold co-exists with alternative conformers (Figure 7A). Nevertheless, mutagenesis of the FSE to disrupt the PK or to favor formation of the alternative folds convincingly demonstrate that the canonical PK is the only conformation driving efficient frameshifting. Recently, an alternative PK was proposed to form in the presence of 5′ extensions, due to the interaction between the SL1 loop and the GGG nucleotides found at the spacer (21,23). Although our chemical probing results cannot definitively exclude the alternative PK fold, our pulling experiments did not detect the steps corresponding to the alternative PK suggesting it is not present in our experimental setup.

Clustering of DMS-MaP reactivities by DREEM analysis detected length-dependent alternative folds at the SARS-CoV-2 frameshift site. In the shorter FSE, FSE-V1 and FSE-V2 variants we detected two distinct conformations. One of the conformations is consistent with the canonical PK fold, whose relative abundance closely matched with the frameshift efficiencies measured in cell-based frameshift assays. The second conformation, which we presumed to be non-frameshift competent one, contained either a shifted SL1 (FSE), a 3WJ (FSE-V1), or a closed SL1 loop that may preclude PK formation (FSE-V2). Strikingly, in the longer FSE-V3 and FSE-V4 variants DREEM analysis detected a single non-PK conformation containing AS1, yet these variants supported higher levels of frameshifting when compared to FSE-V1 and FSE-V2. One possible explanation is that during translation, RNA structures would be unfolded by the helicase activity of the ribosomes allowing the RNA to resample into alternative conformations. Specifically, as the ribosome moves over the 5′ portion of the AS1 stem, it would liberate 3′ part of the stem allowing the folding of SL1, and eventually the formation of the canonical PK fold (Figure 7B). In contrast, structures containing the shifted SL1 cannot frameshift effectively, because helicase disruption of the shifted SL1 would preclude its refolding into the canonical SL1 of the PK. The 3WJ could in principle fold into a PK when base pairing at the slippery sequence is disrupted, as shown by our single molecule pulling data in FSE-A2. On the other hand, in the context of translation, the proximity of the 3WJ to SL1 may reduce the likelihood of the 3WJ refolding into the PK. This can be either due to steric inhibition, insufficient refolding time before the ribosome hits the SL1, or because the SL1 is kinetically trapped and cannot readily refold into the PK conformation. This model would mechanistically explain how high frameshifting efficiencies can be achieved even though the PK is not identified as the dominant structural conformation in genome based structural probing studies (15,22,28). Whilst the PK fold was observed in Cryo-EM structures of SARS-CoV-2 FSE stalled ribosomes (12), these ribosomes were trapped over the SS using a stop codon in place of the second slippery codon (U_UUA_AAC to U_UUA_UAA). Consequently, the 5′ nucleotides occluded by the ribosome would not be base pair into the alternative stem (AS) or three-way junction (3WJ). We therefore predict that ribosomes trapped before the slippery sequence would allow detection of alternative RNA structures, such as the 3WJ conformer, which can be important for fine-tuning SARS-CoV-2 frameshifting for optimal replication.

We also suggest that the presence of less-stable structures can be a strategy employed by RNA viruses to avoid logjams during the replication of their genomes (59,60). In coronaviruses, during the synthesis of the negative strand of the genome, the RNA would be unwound from its 3′ end by the RNA-dependent RNA polymerase, which would first unfold the SL2 before reaching the AS1. This would preclude the formation of the PK structure and allow genome replication without hindrance (10,24,61–63). Thus, less stable folds might be preferred to ensure optimal speed of genome replication at different stages of infection.

Finally, we show that ASO targeting of the SL2 prevents the formation of the canonical PK, thus decreasing frameshifting both *in vitro* and in cells. Nevertheless, the conformational landscape that we identified has implications for therapeutic interventions. So far, efforts to target FSE have mainly focused on the isolated PK element. However, if it is formed transiently during translation or at different stages of the infection cycle, the PK may not be the most abundant structure in the cell. This would impact the efficiency of small molecule targeting. In addition, (de)stabilization of the alternative conformers may lead to a shift in the possible RNA folds, which may ultimately impact frameshifting and reduce viral replication. Knowledge of functionally relevant alternative conformers of viral frameshift elements could therefore be targeted to increase the efficiency of viral RNA targeting. Although our work provides mechanistic insights on structural transitions of FSE variants under defined conditions, future investigations will also be needed to understand the potential role of RNA-binding proteins and longer range cis-interactions on the structure and function of frameshift elements in the context of viral infection in cells.

## DATA AVAILABILITY

Data supporting this study is available at 10.5281/zenodo.6626934.

## CODE AVAILABILITY

Custom scripts were employed to process optical tweezers data. The python algorithm called Practical Optical Tweezers Analysis TOol is available on Github (POTATO, https://github.com/REMI-HIRI/POTATO.git) (35).

## Supplementary Material

gkac1184_Supplemental_File
