# Supplementary material for: Cis-mediated interactions of the SARS-CoV-2 frameshift RNA alter its conformations and affect function
Source: Nucleic Acids Res. 2022 Dec 20;51(2):728–43. doi: 10.1093/nar/gkac1184 (PMC9881162; doi:10.1093/nar/gkac1184)
Supplement: gkac1184_Supplemental_File [file gkac1184_supplemental_file.pdf]

SUPPLEMENTARY FIGURES

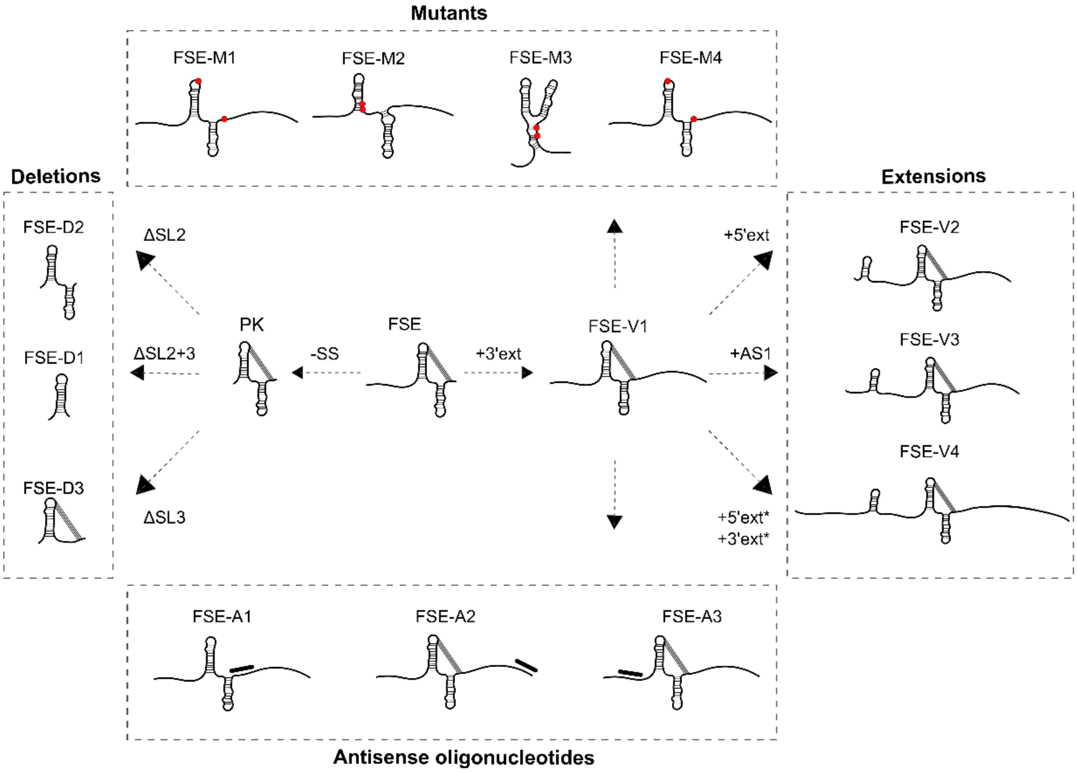

Figure S1. Structural landscape of the SARS-CoV-2 FSE RNA. Schematic depiction of the structural relationship between different RNA variants employed in this study. Related to Figure 1.

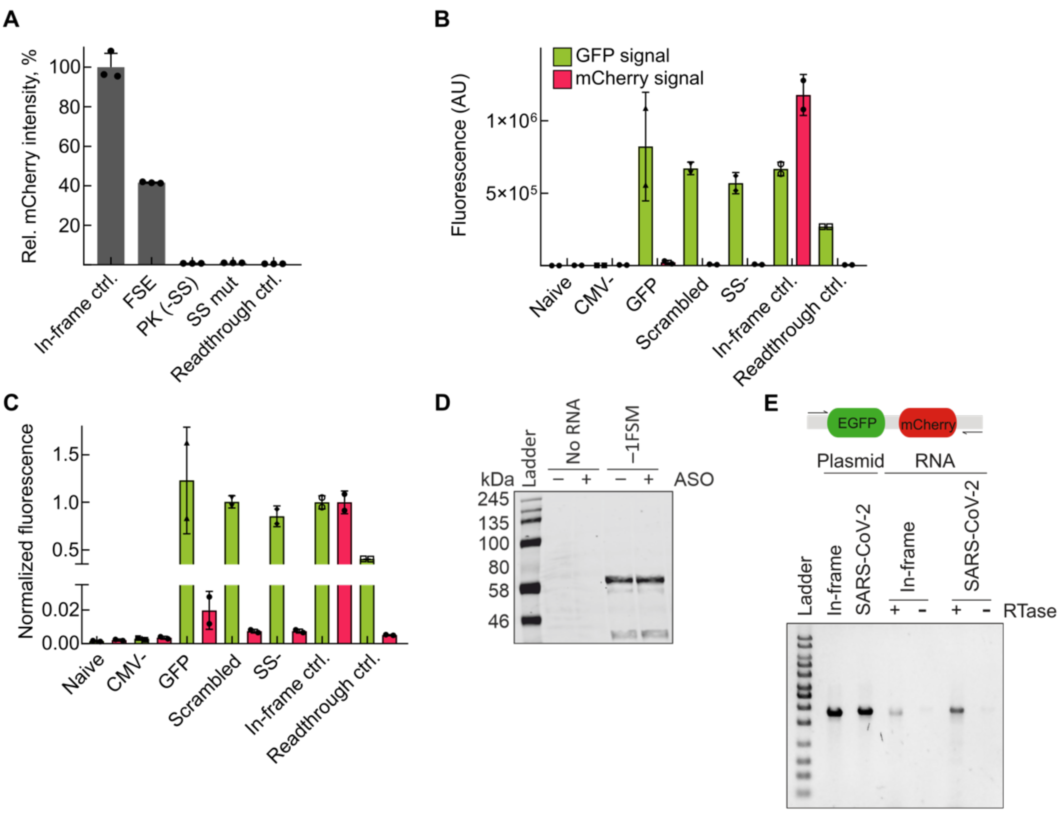

Figure S2. Control experiments of dual-fluorescence assay. (**A**) relative mCherry intensity (analogical to frameshifting efficiency) of selected RNA variants and control samples. FSE contains slippery sequence, spacer and pseudoknot sequence of SARS-CoV-2 RNA. PK(-SS) contains only pseudoknot sequence. SS mut contains mutation in slippery sequence that prevents the frameshifting. Readthrough control contains mCherry gene in 0-frame, thus only upon readthrough event mCherry would be expressed. (**B**) Raw fluorescence values from flow-cytometer for different cell samples. Cells were transfected with no vector (**naïve**), vector lacking CMV promoter (**CMV-**), vector containing only the GFP gene (**GFP**), vector containing GFP in 0-frame, mCherry in –1-frame and the SARS-CoV-2 FSE was scrambled (**scrambled**), vector lacking slippery sequence (**SS-**), vector containing GFP and mCherry genes both in 0-frame without the stop codon in between (**In-frame ctrl.**), vector containing GFP and mCherry genes both in 0-frame with the stop codon in between (**Readthrough ctrl.**). (**C**) Same as B but the values are normalized to the In-frame control. (**D**) In vitro translation experiments in RRL were performed in the presence of –1FS M mRNA, with the mutated frameshift stimulatory element encoding a segment of the in-frame ORF1a-1b. As the control, a non-targeting oligonucleotide was used (“– ASO”). (**E**) Potential splicing of the dual-fluorescence reporter. Schematic depiction of dual-fluorescence reporter construct and position of primer binding sites for PCR amplification of the reporter plasmids and cDNA (**up**). Products of PCR amplification of the in-frame and SARS-CoV-2 frameshift RNA reporter plasmids and oligo(dT)-primed cDNA from cells transfected with the same reporter plasmids are analyzed by using agarose gel electrophoresis (**bottom**).

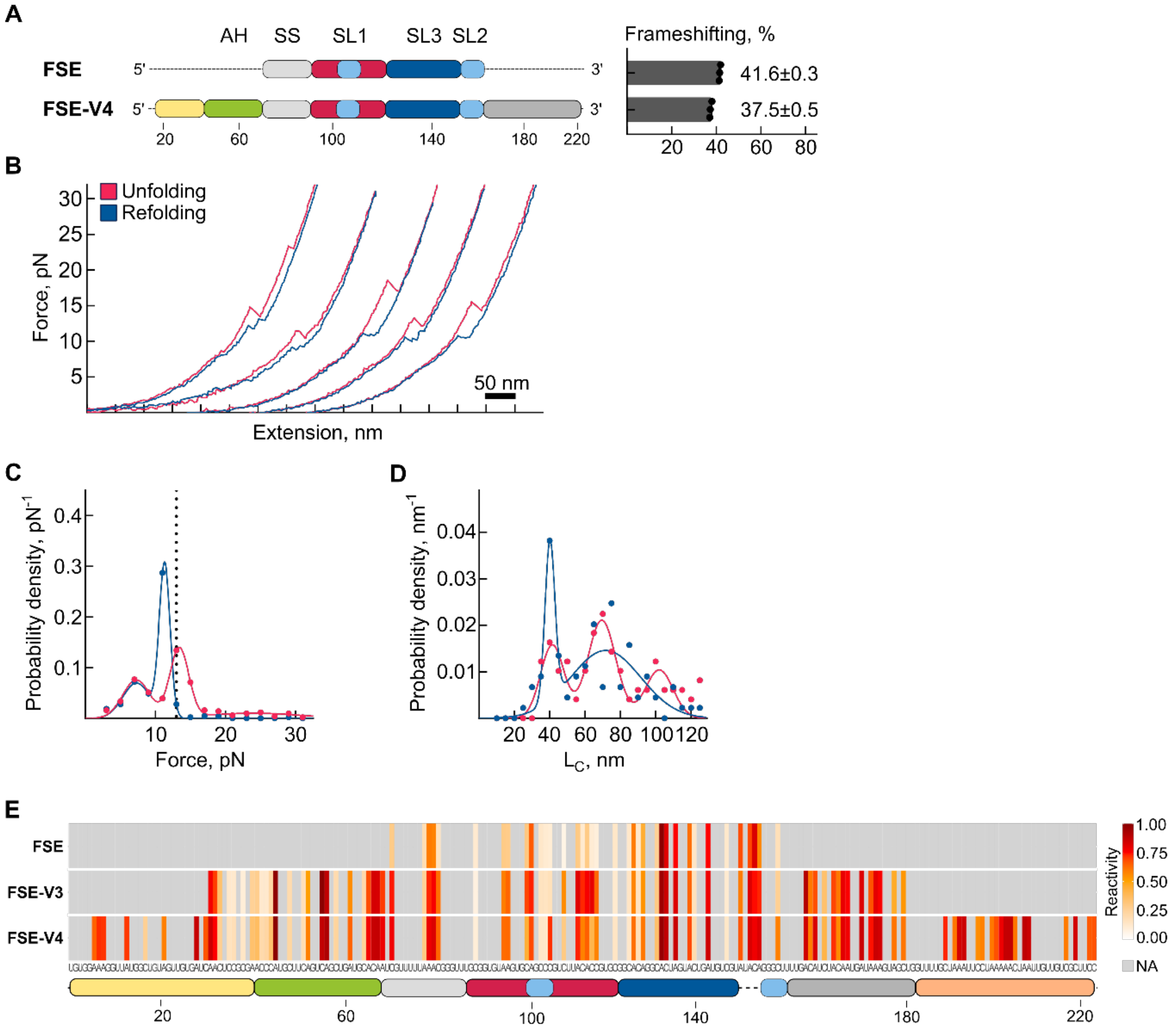

Figure S3. FSE-V4 functional and single-molecule data. (**A**) Schematic representation of RNA variants with respective parts of the FSE employed in the study. Frameshift efficiencies of the variants are shown on the right. (**B**) Example unfolding (red) and refolding (blue) force-distance (FD) curves. The FSE-V4 trajectories exhibit shouldering in the low force regime (<10 pN) resulting in bias of the subsequent fitting. (**C**) Force distribution of the unfolding (red) and refolding (blue) steps observed for the FSE-V4. (**D**) Total contour length distribution of the unfolding (red) and refolding (blue) steps observed for the FSE-V4. (**E**) Reactivity profiles of the RNA variants as determined by DMS-MaP. Related to Figure 2.

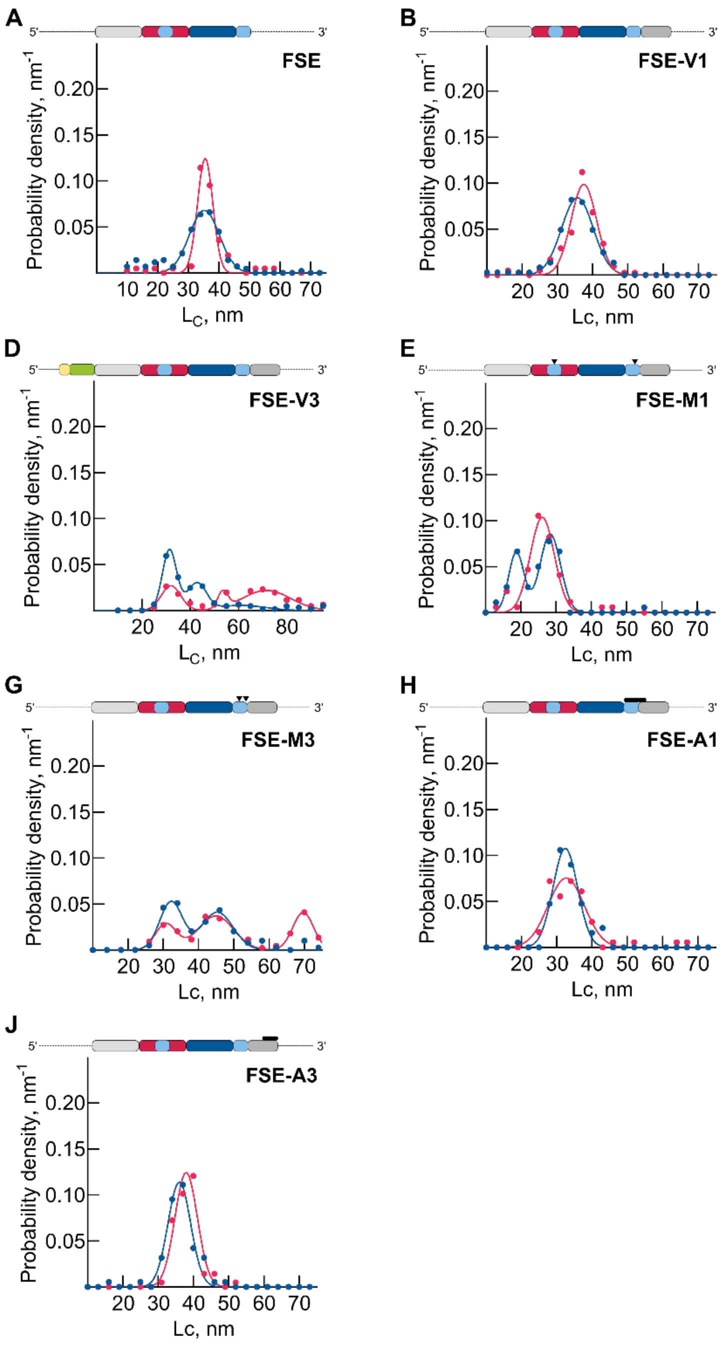

Figure S4. Distributions of (un)folding contour length changes for different RNA variants. (**A**) FSE. (**B**) FSE-V1. (**C**) FSE-V2. (**D**) FSE-V3, (**E**) FSE-M1 (**F**) FSE-M2. (**G**) FSE-M3 (**H**) FSE-A1. (**I**) FSE-A2. (**J**) FSE-A3. Unfolding distributions are in red and refolding are shown in blue. Related to Figures 2-5.

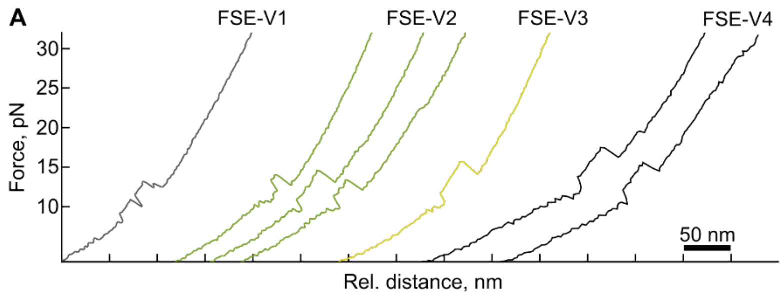

Figure S5. Examples of folding rescue events. (**A**) FD curves showing the folding rescue (indicated by the black arrow) during unfolding for different samples. Related to Figure 2.

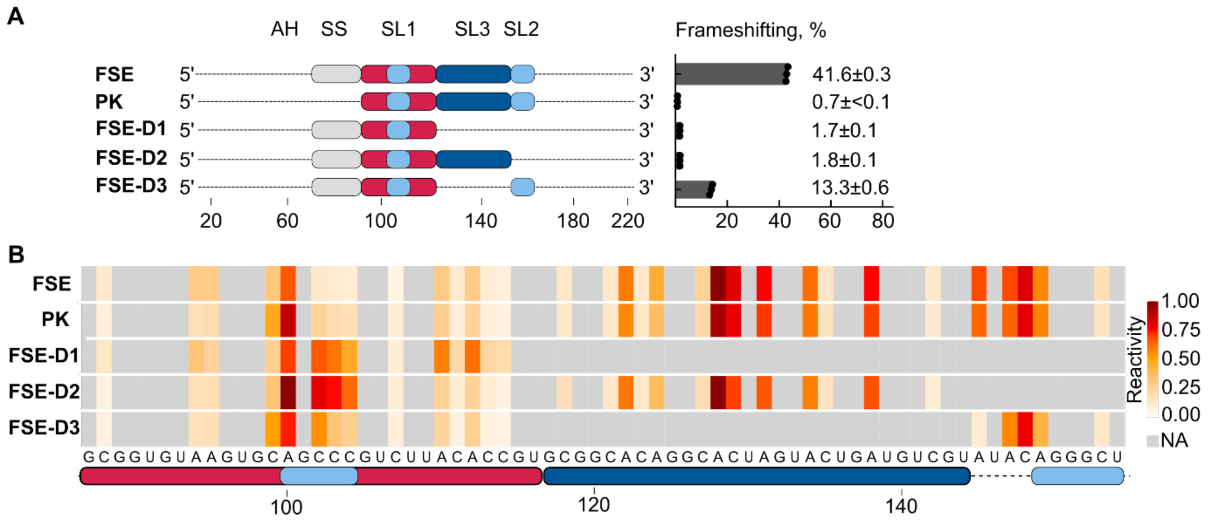

Figure S6. Determination of minimal FS motif. (**A**) A schematic representation of the RNA variants employed with depicted regions of the SARS-CoV-2 FSE and surrounding genomic regions. Frameshifting efficiency (FE) of each RNA as measured by dual-fluorescence assay are indicated at the right. FSE-D1-3 in DF assay contained the slipper sequence. (**B**) Reactivity profiles of the RNA variants as determined by DMS-MaP. Frameshifting values are taken from Zimmer et al., 2021.

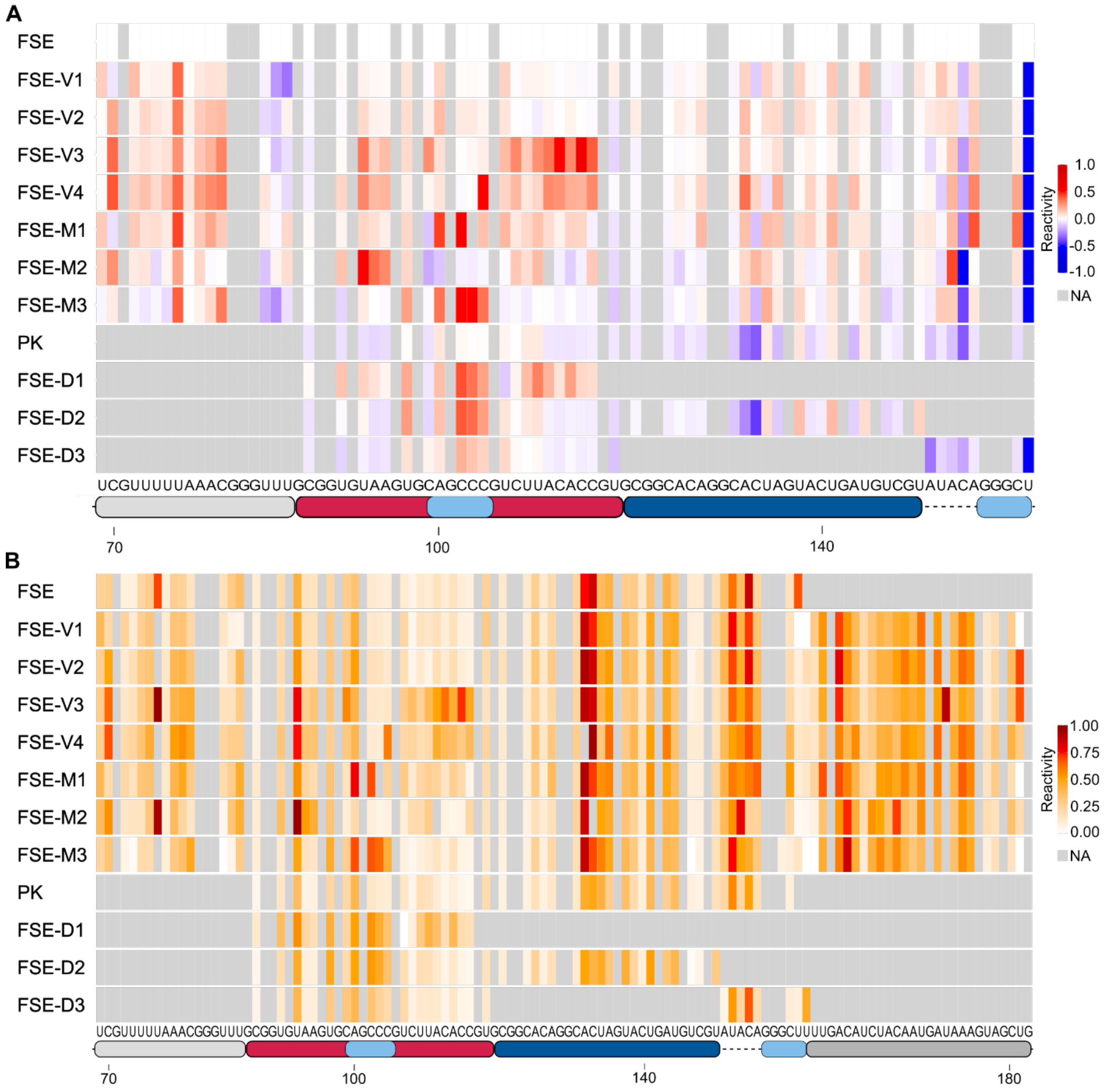

Figure S7. Additional DMS-MaP analysis. (A) Reactivity profiles of the RNA variants as determined by DMS-MaP with U reactivities included. Reactivities of bases were normalized individually within the same base type. **(B)** Differential reactivity profiles of the RNA variants. The values were obtained by subtracting the reactivities of a given base in the FSE sample from the reactivity of a given base in other RNA variants. Related to Figures 2-4.

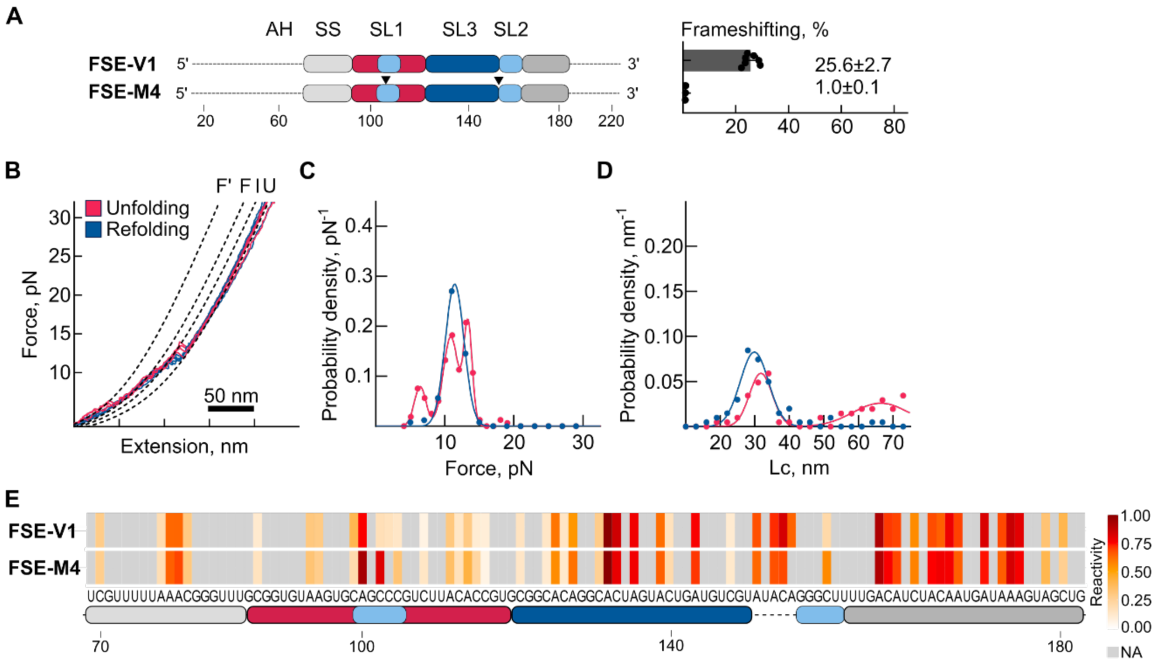

Figure S8. Point mutant stabilizing an alternative pseudoknot. (**A**) Schematic representation of RNA mutants. Frameshift efficiencies of each RNA as measured by the dual-fluorescence assay are plotted at the right. (**B**) Example unfolding (red) and refolding (blue) force-distance (FD) curves. (**C**) Force distribution of the unfolding (red) and refolding (blue) steps observed for the FSE-V4. (**D**) Total contour length distribution of the unfolding (red) and refolding (blue) steps observed for the FSE-V4. (**E**) Reactivity profiles of the RNA variants as determined by DMS-MaP. Related to Figure 2.

SUPPLEMENTARY TABLES

Table S1: Sequences employed to synthesize the RNA variants employed in this study. Nucleotides in small letters indicate the mutations. Slippery site is underlined.

RNA variant	Length	RNA sequence (5’>3’)
FSE	86	UCGUUUUUAAACGGGUUUGCGGUGUAAGUGCAGCCCGUCUUACACCGUGCGGCACAGGCACUAGUACUGAUGUCGUAUACAGGGCU
FSE-V1	114	UCGUUUUUAAACGGGUUUGCGGUGUAAGUGCAGCCCGUCUUACACCGUGCGGCACAGGCACUAGUACUGAUGUCGUAUACAGGGCUUUUGACAUCUACAAUGAUAAAGUAGCUG
FSE-V2	141	CCCAUGCUUCAGUCAGCUGAUGCACAAUCGUUUUUAAACGGGUUUGCGGUGUAAGUGCAGCCCGUCUUACACCGUGCGGCACAGGCACUAGUACUGAUGUCGUAUACAGGGCUUUUGACAUCUACAAUGAUAAAGUAGCUG
FSE-V3	152	AACUCCGCGAACCCAUGCUUCAGUCAGCUGAUGCACAAUCGUUUUUAAACGGGUUUGCGGUGUAAGUGCAGCCCGUCUUACACCGUGCGGCACAGGCACUAGUACUGAUGUCGUAUACAGGGCUUUUGACAUCUACAAUGAUAAAGUAGCUG
FSE-V4	221	UGUGGAAAGGUUAUGGCUGUAGUUGUGAUCAACUCCGCGAACCCAUGCUUCAGUCAGCUGAUGCACAAUCGUUUUUAAACGGGUUUGCGGUGUAAGUGCAGCCCGUCUUACACCGUGCGGCACAGGCACUAGUACUGAUGUCGUAUACAGGGCUUUUGACAUCUACAAUGAUAAAGUAGCUGGUUUUGCUAAAUUCCUAAAAACUAAUUGUUGUCGCUUCC
FSE-M1	114	UCGUUUUUAAACGGGUUUGCGGUGUAAGUGCAGCgCGUCUUACACCGUGCGGCACAGGCACUAGUACUGAUGUCGUAUACAGGGaUUUUGACAUCUACAAUGAUAAAGUAGCUG
FSE-M2	114	UCGUUUUUAAACGGGUUUGCGGUGUAAGUGCAGCCCGUCUUgCcCCGUGCGGCACAGGCACUAGUACUGAUGUCGUAUACAGGGCUUUUGACAUCUACAAUGAUAAAGUAGCUG
FSE-M3	114	UCGUUUUUAAACGGGUUUGCGGUGUAAGUGCAGCCCGUCUUACACCGUGCGGCACAGGCACUAGUACUGAUGUCGUAUACAGGcCcUUUGACAUCUACAAUGAUAAAGUAGCUG
FSE-M4	114	TCGTTTTTAAACGGGTTTGCGGTGTAAGTGCAGCgCGTCTTACACCGTGCGGCACAGGCACTAGTACTGATGTCGTATACtGGGCTTTTGACATCTACAATGATAAAGTAGCTG
PK	67	GCGGUGUAAGUGCAGCCCGUCUUACACCGUGCGGCACAGGCACUAGUACUGAUGUCGUAUACAGGGC
FSE-D1	30	GCGGUGUAAGUGCAGCCCGUCUUACACCGU
FSE-D2	58	GCGGUGUAAGUGCAGCCCGUCUUACACCGUGCGGCACAGGCACUAGUACUGAUGUCGU
FSE-D3	41	GCGGUGUAAGUGCAGCCCGUCUUACACCGUAUACAGGGCUU

Table S2. **Average calculated force values and expected contour length values of different RNA variants employed in this study.** The force and contour length peaks do not always correspond. Related to Figures 2-5.

RNA variant	Direction	Peak	Force, pN	Contour length, nm	Expected contour length, nm	FD curves / molecules
**FSE**	Unfolding	1	11.9±1.8	35.5±2.5	37.2	287/16
		2	20.1±4.9			
	Refolding	1	11.4±1.6	35.3±4.9		
**FSE-V1**	Unfolding	1	13.6±1.6	37.5±3.6	37.2	260/17
	Refolding	1	11.3±0.9	35.7±4.5		
**FSE-V2**	Unfolding	1	12.6±2.1	35.0±2.7	37.2	288/20
	Refolding	1	10.9±1.2	35.7±2.8		
**FSE-V3**	Unfolding	1	5.4±1.1	32.0±4.1	-	244/6
		2	12.7±3.1	53.5±2.1		
		3	-	71.6±10.0		
	Refolding	1	10.4±1.5	31.4±3.0		
		2	-	42.7±3.9		
		3	-	61.2±9.9		
**FSE-V4**	Unfolding	1	7.3±1.9	41.5±6.6	-	187/6
		2	13.4±1.3	69.5±8.0		
		3	24.0±6.6	102.0±7.8		
	Refolding	1	7.3±2.0	40.1±2.8		
		2	11.3±0.8	71.7±19.8		
**FSE-M1**	Unfolding	1	9.4±1.3	26.1±3.4	31.9	118/13
		2	12.6±1.1	-		
	Refolding	1	10.3±1.4	18.7±2.2		
		2	-	28.4±2.9		
**FSE-M2**	Unfolding	1	11.9±1.3	36.3±1.6	37.2	112/8
	Refolding	1	11.6±1.2	36.7±1.9		
**FSE-M3**	Unfolding	1	4.8±0.5	30.7±3.0	65.0	208/17
		2	8.0±2.1	44.9±5.1		
		3	11.3±1.8	69.7±3.0		

	Refolding	1	10.6±1.3	32.2±3.2		
		2	-	45.3±4.5		
**FSE-A1**	Unfolding	1	9.7±0.5	32.7±5.1	31.9	123/10
		2	12.5±1.4	-		
	Refolding	1	11.0±1.5	32.4±3.5		
**FSE-A2**	Unfolding	1	14.9±2.7	35.6±2.4	37.2	206/16
	Refolding	1	11.1±1.2	34.0±2.2		
**FSE-A3**	Unfolding	1	15.0±1.5	37.9±3.2	37.2	132/11
	Refolding	1	11.9±1.3	36.0±3.2		
	Refolding	1	11.5±1.0	21.9±2.7		

Table S3. Step assignments and the percentage of (un)folding events observed at each step. Related to Figures 2-5.

**RNA variant**		**Step #, %**				
		**0**	**1**	**2**	**3**	**>3**
FSE	Unfolding	2.1	72.2	20.8	4.9	-
	Refolding	1.4	26.1	69.0	3.5	-
FSE-V1	Unfolding	-	55.5	40.1	4.4	-
	Refolding	1.6	17.9	74.8	5.7	-
FSE-V2	Unfolding	-	48.6	49.3	2.0	-
	Refolding	0.7	7.9	85.7	5.7	-
FSE-V3	Unfolding	-	19.7	54.9	21.3	4.1
	Refolding	-	12.3	65.6	20.5	1.6
FSE-V4	Unfolding	-	14.3	33.7	31.6	20.4
	Refolding	-	15.7	36.0	40.4	7.9
FSE-M1	Unfolding	-	19.3	80.7	-	-
	Refolding	1.6	37.7	59.0	1.6	-
FSE-M2	Unfolding	-	5.1	69.5	25.4	-
	Refolding	-	5.7	83.0	7.5	3.8
FSE-M3	Unfolding	-	-	28.2	58.2	13.6
	Refolding	-	1.0	44.9	49.0	5.1
FSE-A1	Unfolding	-	5.0	86.7	6.7	1.7
	Refolding	-	9.5	90.5	-	-
FSE-A2	Unfolding	-	82.7	15.4	1.9	-
	Refolding	2.0	15.7	82.4	-	-
FSE-A3	Unfolding	-	65.2	33.3	1.4	-
	Refolding	-	27.0	71.4	1.6	-
